# A Study of the Patient Acceptance Capacity of the Yamanashi Prefecture Medical System amid the Coronavirus Disease 2019 Pandemic

**DOI:** 10.31662/jmaj.2020-0034

**Published:** 2021-01-14

**Authors:** Hiroyuki Kojin, Osamu Inoue, Hiroyuki Kinouchi

**Affiliations:** 1 Department of Quality and Patient Safety, Graduate Faculty of Interdisciplinary Research, Faculty of Medicine, University of Yamanashi, Chuo, Japan; 2 Department of Infection Control, Graduate Faculty of Interdisciplinary Research, Faculty of Medicine, University of Yamanashi, Chuo, Japan; 3 Department of Neurosurgery, Graduate Faculty of Interdisciplinary Research Faculty of Medicine, University of Yamanashi, Chuo, Japan

**Keywords:** Severe acute respiratory syndrome coronavirus 2, COVID-19, Bed occupancy, Pandemics, Medical system

## Abstract

**Introduction:**

Whether healthcare providers can secure the number of beds that may be required during the coronavirus disease 2019 (COVID-19) pandemic remains unclear. This study aimed to determine the sufficiency of the hospital beds available to the healthcare system of Yamanashi, Japan, in accommodating hospitalized and severely ill patients during the COVID-19 pandemic.

**Methods:**

In total, 60 hospitals, with > 20 beds each, were included in this study (*n* = 10,684). However, beds in the psychiatric and tuberculosis wards (*n* = 2,295), nonoperational beds (*n* = 376), and beds for patients in the recovery and chronic phases (*n* = 3,494) were excluded. The projected occupancy rate was calculated based on the estimated number of patients, including severely ill patients requiring hospitalization during the COVID-19 pandemic. Based on the number of hospitalized patients, we created an adjusted model to calculate the mean occupancy rate of beds for each medical area in the prefecture (Model 1), which is free of areal occupancy rate biases. Moreover, we created an adjusted model that places severely ill patients in the two advanced acute hospitals in Yamanashi, thereby calculating the bed occupancy rates in other hospitals with > 200 beds (Model 2).

**Results:**

A total of 4,519 beds were analyzed. Although the existing infectious disease beds may not be able to accommodate the projected number of severely ill patients, the existing capacity can accommodate all patients projected to require hospitalization during the pandemic. In Model 1, the mean bed occupancy rate was 50%. Conversely, in Model 2, advanced acute hospital beds were insufficient for the projected number of severely ill patients, and the mean bed occupancy rate was 72.5%.

**Conclusions:**

Adjustment of patients across the medical area borders enables the existing hospital beds to accommodate the estimated number of patients requiring hospitalization or those who are severely ill.

## Introduction

On December 31, 2019, the Wuhan Municipal Health Commission publicized the results of an epidemiological study conducted on 27 patients with “pneumonia of unknown origin.” ^(1)^ On January 9, 2020, the cause was publicly declared as a new strain of coronavirus ^(2)^, which became known as severe acute respiratory syndrome coronavirus 2 (SARS-CoV-2). With each passing day, the number of patients has increased, and SARS-CoV-2 has infected people worldwide. Human coronaviruses, such as the SARS-CoV and the Middle East respiratory syndrome coronavirus, cause respiratory diseases ^(3), (4)^. SARS-CoV-2 also infects humans and causes a serious, sometimes fatal, pneumonia infection known as coronavirus disease 2019 (COVID-19) ^(5), (6)^. As of March 11, 2020, > 120,000 individuals in 114 countries worldwide were infected. This prompted the WHO to declare COVID-19 a pandemic ^(7)^. In Italy, epicenter of the European Union outbreak, the rise in infections has recently led to a shortage of ventilators and hospital beds in certain regions, thereby causing partial breakdown of the healthcare system ^(8), (9)^.

On January 14 ^(10)^, the first COVID-19 case in Japan was reported, and the first case of person-to-person transmission was confirmed on January 18 ^(11), (12)^. Since then, the number of COVID-19 patients has increased. As of April 25, a total of 12,681 patients have confirmed positive for the virus *via* polymerase chain reaction analysis ^(13)^. In a press conference conducted on February 29, Prime Minister Shinzo Abe requested all designated infectious disease medical institutions to operate at their maximum capacities; this was part of the designed guidelines to increase the infectious disease bed count from 2,000 to 5,000 nationwide ^(14)^. In accordance with this guideline, on March 6, the Ministry of Health, Labour and Welfare’s Headquarters for Novel Coronavirus Disease Control instructed the healthcare systems in all prefectures to prepare for an increased patient load ^(15)^. To facilitate this preparation, the agency published formulas for estimating the number of patients requiring hospitalization as well as those that may require intensive care, respirators, and other special considerations ^(15)^. According to these estimates, the 47 prefectures of Japan will have approximately 2.2 million patients requiring hospitalization. Moreover, even if the 5000 beds outlined in Prime Minister Abe’s guidelines are arranged, the hospitals will only be able to accommodate 2.3% of these patients. Particularly, calculations that depend on mere patient count may not appropriately reflect the reality of the situation due to regional differences in the patient peak timings. Nevertheless, even if the scope of our consideration is limited to one prefecture, it remains unclear whether healthcare providers can secure the number of beds that may be required.

Thus, this study aimed to elucidate whether the number of hospital beds available for the healthcare system in Yamanashi Prefecture, Japan, is sufficient to accommodate the number of hospitalized and severely ill patients during the COVID-19 pandemic. According to a survey conducted by the Japan Medical Association Research Institute, the level of medical resources in Yamanashi Prefecture, i.e., the total number of beds, general beds, doctors, and nurses in the hospital, ranks average among the 47 prefectures in Japan ^(16)^. Thus, an understanding of the situation in Yamanashi will provide a better idea on the national scenario. Moreover, it can help us proactively avoid healthcare system breakdown during patient peak.

## Materials and Methods

The present study was designed as an observational study. The targets of our survey included hospital beds in Yamanashi Prefecture, Japan. Yamanashi ranks 42^nd^ out of Japan’s 47 prefectures in population, with 810,000 inhabitants ^(17), (18)^. It shares a border with Tokyo, Japan’s capital city. In 2018, the Japan Medical Association Research Institute conducted a nationwide survey about the healthcare system in the 47 prefectures in Japan and ranked the level of medical resources in Yamanashi as average across the country ^(16)^. Based on this ranking, examination of the situation in Yamanashi Prefecture will help infer the average situation during the COVID-19 pandemic in Japan.

In this analysis, hospital beds were included if, as of July 1, 2018, they were placed inside a medical institution in Yamanashi Prefecture that possessed ≥20 beds per facility ^(19)^. The hospital beds in the psychiatric and tuberculosis wards were excluded from this analysis. Nevertheless, in accordance with the Infectious Disease Law, the beds for infectious diseases placed in the designated infectious disease hospitals were not excluded. Eventually, the beds that were left unused as of July 1, 2018 were also excluded. Based on reports on hospital bed function that are required pursuant to Article 30-12 of the Medical Care Act,**the hospital beds were categorized into four phases: advanced acute, acute, recovery, and chronic ^(20)^. The recovery-phase beds are primarily used for rehabilitation, whereas the chronic-phase beds are used by patients requiring long-term recuperative care. Thus, beds categorized as either recovery-phase or chronic-phase were also excluded from this analysis.

Hospitals with the target beds were categorized into two: those with >200 beds and those with <200 beds. Hospitals with >200 beds can reasonably be assumed to possess the doctors, nurses, and other human resources required for administering acute-phase care.

Yamanashi Prefecture is geographically divided into four medical areas: the mid-north area, in which the prefectural capital of Kofu is located, the East-Valley area, the South-Valley area, and the Mt. Fuji-East area. Because of uneven geographic distribution of hospitals that possess the target beds, an analysis that separately considers each medical area was also conducted.

To calculate the patient load ^(21)^, the following equation, published by the Japanese government and supported by Nishiura et al. for estimating the daily count of newly hospitalized COVID-19 patients during the peak infection periods ^(15)^, was used ^(22)^:

(*population aged* 0–14)×0.05/100+(*population aged* 15–64)×0.02/100 + (*population aged* ≥65)×0.56/100

Furthermore, the following equation, also published by the government, was applied to calculate the daily number of new severely ill patients requiring intensive care, respirators, and other special considerations during the COVID-19 pandemic ^(15), (21), (22)^:

(*population aged* 0–14) × 0.002/100+(*population aged* 15–64) × 0.001/100 + (*population aged* ≥65) × 0.018/100

This equation was estimated using a mathematical model based on the COVID-19 epidemic scenario, as of February 29, 2020, to calculate the standards for ensuring an appropriate healthcare system in each prefecture ^(23)^. Because the proportion of patients who were hospitalized and severely ill varied with age, the patients are divided into three age groups: 0–14 years, 15–64 years, and ≥65. The coefficients for each age group were calculated based on a survey conducted on all 38,818 people in China from December 8, 2019, to February 1, 2020 ^(24)^. Therefore, it is important to consider the possibility of deviation from the model in Japan. In this study, we determined whether the use of such equation is appropriate based on the fact that the Ministry of Health, Labour and Welfare informed the prefectures on March 6, 2020, that they should review their healthcare systems based on this equation.

The number of projected patients in each medical area was distributed per the population ratio for each medical area calculated using the census data published by Yamanashi Prefecture on February 1, 2020 ^(25)^. While the rates of aging of each medical area remain nonidentical, areas with higher aging rates are uniformly and less densely populated, thus making it unlikely for infection clusters to occur. Thus, adjustment for the aging rates was not performed.

After obtaining the number of patients requiring hospitalization, the occupancy rate (%) of the target beds was calculated. Then, hospitals with <200 beds were excluded, and the occupancy rate (%) of beds in hospitals with ≥200 beds was calculated. These steps were repeated using the estimated number of severely ill patients; the occupancy rates (%) of all infectious disease beds and infectious disease beds in hospitals with ≥200 beds were calculated.

Moreover, to avoid saturation, indicated by occupancy rates >100%, adjusted models were constructed. In Model 1, the patient loads were adjusted across hospitals with ≥200 beds to achieve occupancy rates that are as close as possible to the areal mean. In Model 2, severely ill patients were placed in the two advanced acute hospitals, located in the mid-north area. The remaining patients requiring hospitalization were adjusted across all other hospitals with ≥200 beds to achieve occupancy rates that are as close as possible to the areal mean. The allocation of severely ill patients in Model 2 was set based on the plan for securing hospital beds in Yamanashi Prefecture as of April 16 ^(26)^.

Based on the above considerations of Models 1 and 2, we also considered whether medical equipment and personnel at medical institutions accepting severely ill patients could cope with the situation. To determine the number of required medical devices and clinical practitioners, we used the results from an emergency survey, released on March 9 and conducted by the Japanese Society of Respiratory Care Medicine and the Japan Association for Clinical Engineers ^(27)^. Moreover, we considered the number of intensive care units required for severely ill patients. This information was based on the results of the FY 2017 Reports on Medical Functions of Hospital Bed ^(28)^.

Models were created using Microsoft Excel 2016.

## Results

**Figure 1.**
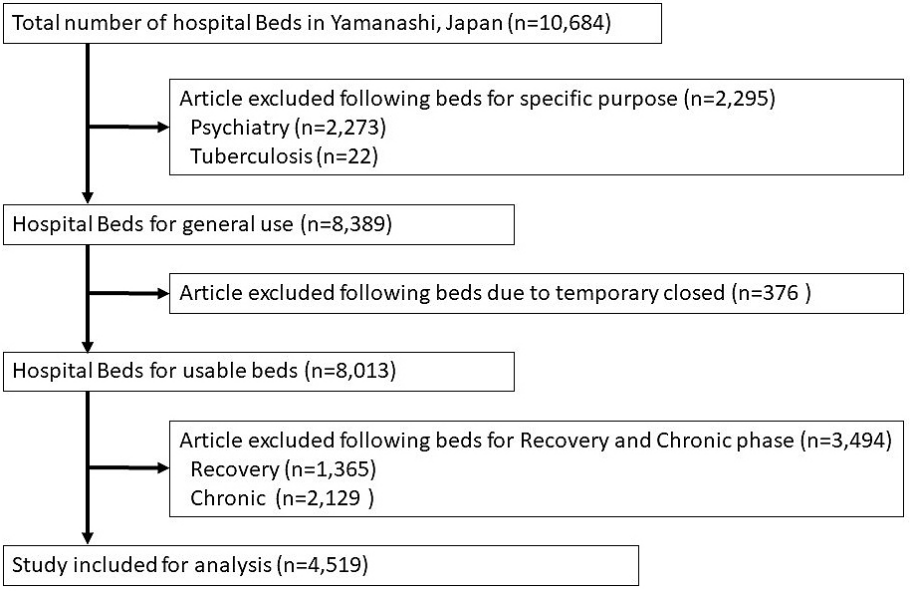
Study identification, inclusion, and exclusion criteria.

Yamanashi prefecture has a total of 60 hospitals and 10,684 hospital beds (Figure 1). After excluding specific-purpose beds (*n* = 2,295), nonoperational beds (*n* = 376), and recovery- and chronic-phase beds (*n* = 3,494), a total of 4,519 beds were analyzed.

Table 1 presents the number of beds in each bed category, infectious disease bed count, total hospitals, and population distribution for each of the four medical areas of Yamanashi (Table 1). Yamanashi Prefecture comprises 1,149 advanced acute beds. Of the 66 beds, 49 advanced acute beds in the Mt. Fuji-East area are located in a hospital with less than 100 beds that specializes in orthopedic surgery and neurosurgery. By excluding these 49 beds, considered unsuitable for the treatment of infectious diseases, 17 advanced acute beds are left in the Mt. Fuji-east area, bringing the total number of beds in Yamanashi Prefecture to 1,100, 98.5% of which are placed in the mid-north area. Contrarily, of the 3,370 acute-phase beds in the prefecture, approximately 50% are placed in the mid-north area; the East-Valley and Mt. Fuji-East areas have approximately 20% each, and the South-Valley area has <10%. Each area has infectious disease beds, including a total of 28 beds across 7 hospitals, comprising 0.6% of all target beds.

**Table 1.** Number of Beds in Each Function And Medical Area.

	Medical area								
	Mid-North	%	East-Valley	%	South-Valley	%	Mt. Fuji East	%	Total
Number of all beds category	2,746	60.8	727	16.1	307	6.8	739	16.4	4,519
Advanced acute phase^*^	1,083	94.3 (98.5)	none	-	none	-	66 (17)	5.7 (1.5)	1,149 (1,100)
Acute phase	1,663	49.3	727	21.6	307	9.1	673	20.0	3,370
Beds for designated infectious diseases	12	42.9	4	14.3	4	14.3	8	28.6	28
Number of hospitals	18	47.4	9	23.7	5	13.2	6	15.8	38
>=200 beds	6	66.7	1	11.1	none	-	2	22.2	9
200 beds <	12	41.4	8	27.6	5	17.2	4	13.8	29
Resident population	457,781	56.5	130,798	16.1	48,056	5.9	173,382	21.4	810,017

*The number of parentheses excludes 49 beds in a hospital with less than 100 beds in the Mt. Fuji east area.

In total, 38 hospitals throughout the prefecture have the target beds for this analysis, and 29 of them (76.3%) have <200 target beds each. The South-Valley area is home to zero medical institutions with ≥200 target beds, whereas the East-Valley is home to one medical institution. Approximately 56% of Yamanashi’s permanent population resides in the mid-north area, whereas the South-Valley area is home to only about 6%.

Table 2 presents the occupancy rates of target beds based on the calculated estimates of the number of severely ill patients and those requiring hospitalization. It also presents the results of our adjusted models. Throughout the COVID-19 pandemic in Yamanashi, an estimated number of 1,530 patients require hospitalization, and an estimated number of 50 individuals are classified as severely ill patients ^(21)^. The breakdown of patients requiring hospitalization by the medical area: 865 patients in the mid-north area (56.5%), 327 in the Mt. Fuji-East area (21.4%), 247 in the East-Valley area (16.1%), and 91 in the South-Valley area (5.9%). Moreover, the breakdown of severely ill patients: 28 in the mid-north area (56.0%), 11 in the Mt. Fuji-East area (22.0%), 8 in the East-Valley area (16.0%), and 3 in the South-Valley area (6.0%).

**Table 2.** Occupancy Rates of Hospitalization and Severely Ill Patients in Medical Areas.

	Medical Area				
	Mid-North	East-Valley	South-Valley	Mt. Fuji East	Total
Requiring hospitalization (person)	865	247	91	327	1,530
Number of total beds	2,746	727	307	739	4,519
Occupancy rate (%)	31.5	34.0	29.6	44.2	33.9
Only >=200 hospitals (beds)	2,401	293	none	575	3269
Occupancy rate (%)	36.0	84.3	>100	56.9	46.8
Severely ill patients (person)	28	8	3	11	50
Number of beds for infectious diseases	12	4	4	8	28
Occupancy rate (%)	233.3	200.0	75.0	137.5	178.6
Only > =200 hospitals (beds)	8	4	none	4	16
*Occupancy* rate (%)	350	200	>100	275	312.5
Model 1					
Adjusted patients requiring hospitalization (person)	1,095	147	0	288	1,530
Increase or decrease	+230	-100	-91	-39	
Only > =200 hospital					
Occupancy rate (%)	45.6	50.2	-	50.1	46.8
Model 2					
Adjusted severely ill patients (person)	50	none	none	none	50
Increase or decrease	22	-8	-3	-11	
Only > =200 hospital					
without two advanced acute hospital					
Number of beds	1,242	293	none	575	2,110
Adjusted patients requiring hospitalization (person)	901	212	-	417	1,530
Increase or decrease	+36	-35	-91	+90	
Occupancy rate (%)	72.5	72.5	-	72.5	72.5

Model 1: Adjusted the number of patients to the same bed occupancy rate in all areas. Model 2: Adjusted the patients with severe condition to the two advanced acute hospitals in the mid-north area and the patients requiring hospitalization to another hospital with more than 200 beds.

The bed occupancy rate of patients requiring hospitalization was determined to be 33.9% of all the target beds. These occupancy rates ranged from 29.6% to 44.2% across the medical areas. Thus, a comparison of all the target beds indicates the projected patient count requiring hospitalization. Contrarily, in terms of the bed occupancy rates of hospitals with >200 beds, the South-Valley area is completely saturated due to the lack of such hospitals, and the East-Valley area is highly occupied, with an occupancy rate of 84.3%.

The occupancy rate of severely ill patients is 178.6% of the total number of infectious disease beds available throughout Yamanashi, thus indicating the total saturation of the medical system. With regard to the areal occupancy rates, the South-Valley area is the only area with a rate of <100%, at 75%; however, the rates of the other three areas range from 137.5% to 233.3%, all of which exhibit saturation. Furthermore, if only infectious disease beds in hospitals with >200 beds are considered, the South-Valley area, which has no such beds, also becomes saturated. Moreover, the three other areas exhibit bed occupancy rates ranging from 200% to 350%.

In Model 1, patients requiring hospitalization were adjusted to allow bed occupancy rates at hospitals with >200 beds to achieve the nearest areal mean rates. If each of the three other areas sends 230 patients to the mid-north area, the bed occupancy rates of each area will be approximately 50%. Likewise, in Model 2, 22 severely ill patients located in the three other areas were sent to the two advanced acute hospitals located in the mid-north area. Thus, after excluding the beds in the two hospitals of the mid-north area that houses severely ill patients, the bed count in hospitals with ≥200 beds reduced from 2,401 to 1,242. The adjustment of the patient count in each area to achieve a uniform areal mean requires the East-Valley area to send approximately 35 patients to the mid-north area and the South-Valley area to send approximately 90 patients to the Mt. Fuji-East area. This enables all medical areas to exhibit an occupancy rate of 72.5%.

According to the results of an emergency survey conducted by the Japanese Society of Respiratory Care Medicine and the Japan Association for Clinical Engineers ^(27)^, the total number of ventilators in Yamanashi Prefecture was 208, of which 126 were on standby as of March 9. Moreover, there were 10 units of extracorporeal membrane oxygenation (ECMO), 9 of which were on standby. The same survey indicates that there is a total of 115 clinical engineers employed by hospitals in Yamanashi Prefecture as of March 9.

According to the results of the FY 2017 Reports on Medical Functions of Hospital Beds ^(28)^, in Yamanashi Prefecture, there were 3 ICUs and 28 ICU beds, of which one ICU with six beds was located in the Mt. Fuji-East area; two units had 10 and 12 beds in two advanced acute hospitals, placed in the mid-north area.

## Discussion

This investigation on the rates of hospital bed occupancy in the healthcare system of Yamanashi Prefecture, which ranks at or near the mean level of medical resources available throughout Japan, reveals that the existing capacity of the healthcare system is sufficient to accommodate the projected number of patients requiring hospitalization during the COVID-19 pandemic. Contrarily, the existing number of infectious disease beds makes it impossible to accommodate the projected number of severely ill patients requiring the repurposing of beds in advanced acute hospitals. For an estimated number of 50 severely ill patients, there were 126 ventilators on standby as of March 9, and a maximum of 115 clinical engineers could be secured. Meanwhile, as of March 9, there were nine ECMO units on standby, and approximately 20% of severely ill patients were considered to be at the upper limit of demand. For an estimated number of 50 severely ill patients, the number of ICU beds in Yamanashi Prefecture was 28. Thus, this divergence clearly indicates that care for severely ill patients needs to be provided by facilities other than ICUs. Particularly, in the distribution of severely ill patients in Model 2, there are only 10 and 12 ICU beds in each of the two advanced acute hospitals. Thus, if 25 patients are accepted in each model, 13–15 patients will always need to be managed outside the ICU. This is a major challenge to staffing and equipment allocation and thus strongly suggests the requirement of preventive measures to curb the incidence of severely ill patients.

In 2015, the number of hospitalized beds in Japan was 13.2 per 1,000 people compared with 4.9 in an average of other Organization for Economic Co-operation and Development (OECD) countries ^(29)^. This study revealed that the hospital beds available during the COVID-19 pandemic in Yamanashi are able to avoid saturation through regional coordination, despite the high occupancy rate. The reason for this disparity is that Japan has the largest number of beds per 1000 people in the OECD.

Infectious disease hospitals were established in accordance with Article 38 of the Act on the Prevention of Infectious Diseases and Medical Care for Patients with Infectious Diseases. In Japan, a total of 410 hospitals with 1871 beds were established. In Yamanashi ^(30)^, 7 hospitals with 28 beds were established and were expected to be a crucial part of the first line of defense against new infectious diseases. About three of these facilities are small- to medium-scale medical institutions with <200 beds ^(19)^. Thus, only 4 hospitals with >200 beds each, collectively housing a maximum of 16 beds, can be used to provide infectious disease treatments to severely ill patients requiring the use of significant healthcare resources. Thus, as presented by the results of the adjusted model constructed in this study, the plans for interareal medical exchange are warranted.

An adjusted model in which all patients hospitalized for COVID-19 are placed in hospitals with ≥200 beds has estimated that approximately 50% of such beds will be occupied throughout the prefecture. This indicates that 50% of the normal capacity of the hospital care system will be effectively reduced. While some standby surgeries or educational hospitalization can be waived off, certain individuals, such as cancer patients or patients with emergency conditions, require prompt hospitalization. In Yamanashi, the “Yamanashi Action Plan for Pandemic Influenza and New Infectious Diseases” was enacted on February 4, 2014, and was revised twice, that is, in 2018 and 2019 ^(31)^. The Action Plan indicates the need for business continuity management *via* a business continuity plan (BCP), which needs to be primarily managed by administrative agencies. In particular, during the spread of a new infectious disease, the continuation of the healthcare system entirely depends on the BCPs individually developed by the medical institutions ^(31)^. Under this system, the adjustment of the healthcare services inside a medical area and the healthcare provision between medical areas seem impossible. The results of this research indicate that if advanced acute hospitals are burdened with care of severely ill patients, then 72.5% of the beds in hospitals with >200 beds will be occupied by COVID-19 patients. In such circumstances, the normal capacity of hospitals providing care will be significantly limited, and a medical surge (an emergency situation in which medical need outperforms the accommodative and responsive capacity of healthcare services) is likely possible.

In 2015, the “Yamanashi Infectious Disease Network” commenced its operations, along with the involvement of the Yamanashi Health Promotion Division, Director of Public Health Centers, Yamanashi Prefectural Central Hospital, Association of Infection Control Nurses, Japan Pediatric Association, and University of Yamanashi, thereby including individuals from various professions, such as doctors, nurses, public health nurses, pharmacists, clinical technicians, and administrative officials ^(32)^. While this network was intended to serve as a platform for medical exchange inside the prefecture during the emergence of a new infectious disease, currently, it lacks sufficient participation of the stakeholders of medical exchange services. This prevents the network from fulfilling its role. During the COVID-19 pandemic, the core members of this network worked collaboratively to create a specialist council for medical exchange inside Yamanashi ^(33)^. They are presumed to have the ability to develop a framework that allows medical exchange not only during crisis but also during normal times.

Similarly, a wide-area transport across the prefectures is also a challenge. In late April, >90% of hospital beds for COVID-19 patients in Tokyo were filled ^(34)^. Simultaneously, it was estimated that 150 of the 200 beds in the ICU that were redirected to COVID-19 patients were already in use ^(35)^. At this time, the hospitals in Yamanashi Prefecture had sufficient capacity to accommodate COVID-19 patients. In this study, we examined the movement of patients across medical areas in Yamanashi Prefecture. Such movement is expected to reduce the saturation of beds. As prefectural governments are the main body for infectious disease control, the involvement of the central government is necessary to achieve successful transfer of patients across prefectures.

The present study has several limitations. First, the occupancy rate was calculated based on the assumption that all hospitals are operating normally. In practice, it is assumed that the medical surge will worsen due to dropout of medical personnel or deterioration of hospital functions caused by the occurrence of hospital-acquired infection ^(36)^. The results of this study indicate that it is important to operate the healthcare system as normal as possible to avoid medical surges during the COVID-19 pandemic. Second, as this study focused on hospitalization and beds during the COVID-19 pandemic, it did not consider outpatient care services. Outpatient care, such as visits for fevers, will also require patient care and other medical resources, which is presumed to overwhelm the activity of the prefectural hospitals. Taking this into consideration, it may be practical for hospitals with unused beds to provide hospitalized care, hospitals with <200 beds, and hospitals specializing in recovery- and chronic-phase beds to fully contribute to this work front, to prevent a medical surge. Finally, this study did not make special considerations for children, pregnant mothers, and other populations with specific needs. Furthermore, research on the estimates of the number of COVID-19 patients requiring special considerations and a more comprehensive analysis of the functions of the healthcare system are warranted.

## Article Information

### Conflicts of Interest

None

### Acknowledgment

We would like to thank the support provided by Ms. Shiho Amagasa of the Department of Preventive Medicine and Public Health, Tokyo Medical University. We also thank Editage (http://www.editage.com) for editing and reviewing this manuscript for English language.

### Author Contributions

All authors were involved in drafting the article or revising it critically for important intellectual content and have read and approved the final version of the manuscript. Hiroyuki Kojin was responsible for the design, analysis, interpretation, and drafting of the manuscript. Osamu Inoue and Hiroyuki Kinouchi revised the manuscript.

### Approval by Institutional Review Board (IRB)

The approval was not required in our research.
